# A Rare Presentation of Isolated CNS Posttransplantation Lymphoproliferative Disorder

**DOI:** 10.1155/2017/7269147

**Published:** 2017-01-02

**Authors:** Jaime Morris, Casey Smith, Andrew Streicher, Allison Magnuson, Susan Newman, Robert Bertoli

**Affiliations:** ^1^University of Tennessee Graduate School of Medicine, 1924 Alcoa Highway, P.O. Box U-94, Knoxville, TN 37920, USA; ^2^College of Medicine, The University of Tennessee Health Science Center, 920 Madison Avenue, Memphis, TN 38163, USA; ^3^University Cancer Specialists, 1926 Alcoa Highway, Suite F-350, Knoxville, TN 37920, USA; ^4^University Radiation Oncology, 1926 Alcoa Highway, Suite 130, Knoxville, TN 37920, USA

## Abstract

Posttransplantation lymphoproliferative disorder (PTLD) is a recognized and extremely morbid complication of solid organ transplantation, but central nervous system involvement, particularly in isolation, is rare. There are no standardized treatment strategies for PTLD, though commonly used strategies include reduction of immunosuppression, chemotherapy, rituximab, radiation, and surgery. We present a case of an unusual morphologic variant of primary central nervous system PTLD with successful response to rituximab and cranial radiation. A 69-year-old Asian male, who underwent postrenal transplant nine years earlier, presented with a one-month history of new onset seizure activity. His evaluation revealed multiple brain lesions on magnetic resonance imaging (MRI), as well as serologic and cerebrospinal fluid studies which were positive for Epstein-Barr Virus (EBV) infection. Ultimately, he underwent craniotomy with tissue biopsy with the final pathology report showing posttransplant lymphoproliferative disorder, polymorphic type. The patient was managed with reduction in immunosuppression, rituximab therapy, and cranial radiation treatments. He had demonstrated marked improvement in his neurologic function and was ultimately discharged to inpatient rehabilitation facility.

## 1. Introduction

Immunosuppression is a key component to avoiding allograft rejection in transplantation. Posttransplantation lymphoproliferative disorder (PTLD) is not only a recognized complication but also one of the most common malignancies of solid organ transplantation with an incidence between 1% and 3% in transplant recipients. Incidence varies based on patient age, organ type transplanted, and type of immunosuppression. Ghobrial et al. quote a median age of diagnosis at 48 years (range, 15 to 75 years), with only 22% of the study group greater than 60 years old [1]. Liver and kidney transplants were the most common type associated with PTLD and the median time from solid organ transplantation (SOT) to diagnosis was 19 months (range, 0 to 206 months) [1]. PTLD is a spectrum of heterogeneous lymphoid proliferations often driven by Epstein-Barr Virus (EBV) infections. The most common form, systemic PTLD, displays diverse morphology, often resembling diffuse large B-cell lymphoma. Primary central nervous system (PCNS) involvement is a rare form of PTLD, especially in isolation, comprising 5–15% of all PTLD cases [2]. In comparison to systemic PTLD, isolated CNS PTLD often has a delayed presentation with a typical time from SOT to diagnosis of four to five years [3, 4]. CNS disease has previously been characterized by multifocal lesions, most commonly in the cerebral hemispheres involving subcortical white matter or basal ganglia. Isolated CNS PTLD is typically monomorphic and consistently more aggressive than its systemic counterpart [5]. Long-term prognosis of central nervous system PTLD is considered extremely poor [3, 4, 6, 7].

## 2. Timeline

See Figure 1.

## 3. Case Presentation

Our patient is a 69-year-old Asian male who was admitted with acute encephalopathy and acute respiratory failure secondary to left frontal lobe parenchymal hemorrhage with surrounding edema following a witnessed seizure. Just prior to this admission, he was having episodes of confusion, twitching, and mispronouncing words (aphasia versus dysarthria). Approximately one month prior to admission, patient had an episode of dizziness without syncope while walking outside. He lowered himself to the ground and then went unresponsive with witnessed seizure activity. Initial work-up at out-of-state hospital included computed topography of brain which demonstrated no acute intracranial findings and magnetic resonance imaging (MRI) of his brain which demonstrated multiple brain lesions. He was started on anticonvulsant therapy with levetiracetam. An intensive evaluation for infectious processes was completed, including serologic and cerebrospinal fluid studies which were positive for Epstein-Barr Virus (EBV) (Table 1).

**Figure 2:**
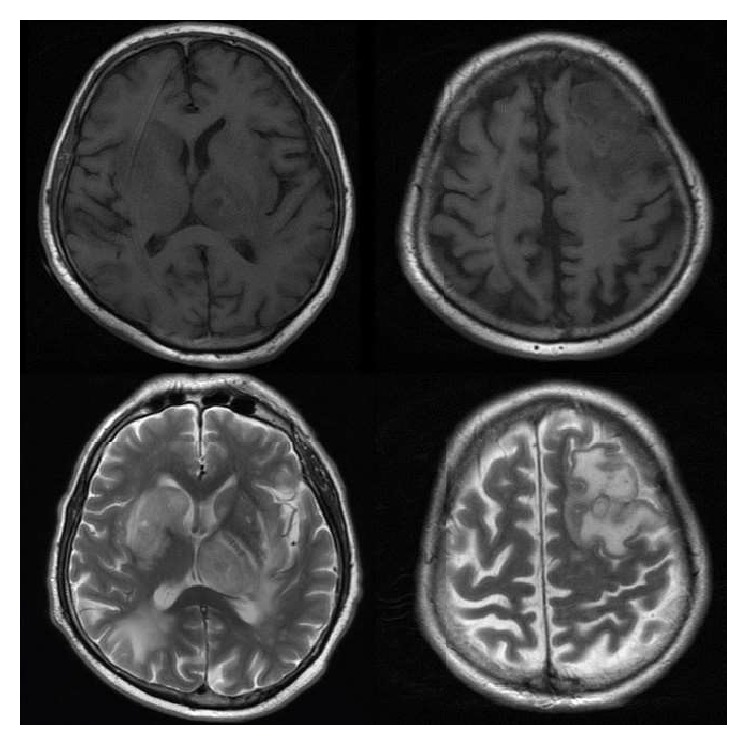
MRI at admission (day 34). Top two images are T2-weighted, middle two are T1-weighted, and bottom two are T2/FLAIR-weighted. Multifocal enhancing lesions in both cerebral hemispheres and in the left cerebellum concerning metastasis, infection, or inflammatory etiology. Redemonstration of areas of hemorrhage in the left frontal lobe is seen on CT scan. 4 mm left to right midline shift is present. Centrally necrotic lesion in the left thalamus measures 14 × 11 mm. Lesion in the right caudate head measures 11 × 10 mm. Enhancing lesion in the posterior left cerebellum measures 9 × 6 mm.

His past medical history is significant for end-stage renal disease with a two-year history of hemodialysis and live donor kidney transplant in 2007 (nine years ago), hypertension, diet-controlled diabetes mellitus, diastolic heart failure/resolved high output heart failure status postligation arteriovenous fistula, and hypothyroidism. On admission, his immunosuppression regimen was mycophenolate mofetil (CellCept) 750 mg twice a day, tacrolimus (Prograf) 0.5 mg every 12 hours, prednisone 5 mg daily, and prophylactic sulfamethoxazole 800 mg/trimethoprim 160 mg three days a week. He was a former smoker, with no history of alcohol or illicit drug use. His temperature was 97.4 F, blood pressure 130/71 mmHg, pulse rate 66 bpm, and respiratory rate 20 breaths per minute. Lung fields were clear on auscultation; cardiac exam demonstrated normal heart sounds and no murmurs. Initial neurological exam was limited following paralytics and sedation for intubation, but he did not move his right extremities to pain. Repeat head imaging demonstrated multifocal parenchymal hemorrhage with surrounding cerebral edema and mass effect with a 5 mm left to right shift (Figure 2).

**Figure 3:**
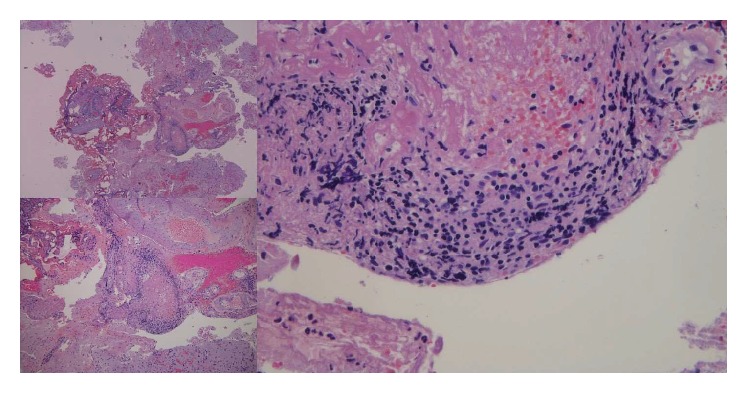
Brain biopsy pathology (day 50). Brain tissue fragments with hematoxylin and eosin stain showing blood vessels surrounded by predominantly mononuclear cells. Occasional thrombi are present as well as fibrinoid and liquefactive necrosis. No granulomas or multinucleated cells are seen.

**Figure 4:**
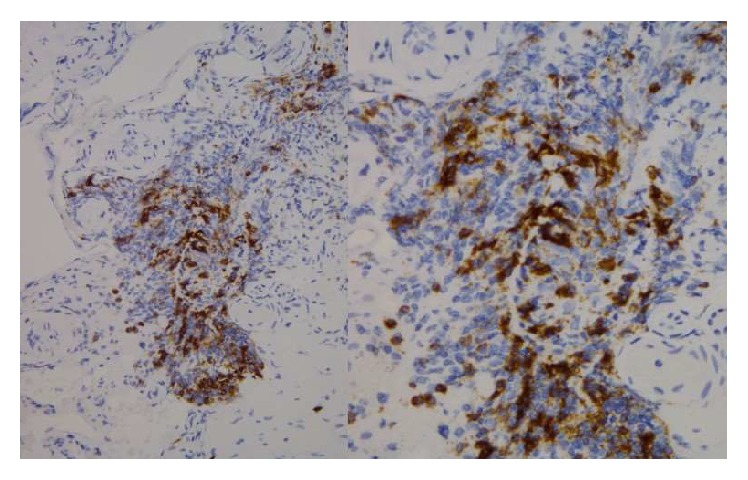
Immunohistochemistry/CD20 stain of brain biopsy (day 50). CD20+ perivascular B-cell infiltrates. Many also are staining positive for EBV-encoded RNA (EBER), which is not depicted but indicates presence of Epstein-Barr Virus. Additional stains of the sample suggested increased presence of polymorphic plasmacytes and T-cells.

Given his history of renal transplant and immunosuppression, the differential diagnosis included malignancy (lymphoma) versus infection. He was treated with broad spectrum antibiotics and antiviral agents. The brain lesions were not in ideal location for brain biopsy, so patient underwent full body positron emission tomography (PET) to look for other lesions/sites that were more amenable to biopsy. PET scan revealed the lesions were confined to the central nervous system. From his initial presentation with vague systems of confusion and inappropriate speech, the patient progressed to grand-mal seizures. Patient continued to deteriorate with increasing confusion, apraxia, gaze deviation, and flaccid hemiparesis. Given the progression of symptoms, vasculitis versus infection versus CNS lymphoma was considered likely candidates. Without a clear diagnosis of the brain lesions, the patient underwent right frontal craniotomy for tumor resection and tissue biopsy with stealth frameless stereotaxy and use of operating microscope. Intraoperative tissue biopsy showed atypical perivascular lymphoid infiltrate, more consistent with a vasculitis (Figures 3 and 4). However, the final pathology report confirmed posttransplant lymphoproliferative disorder, polymorphic type.

**Figure 5:**
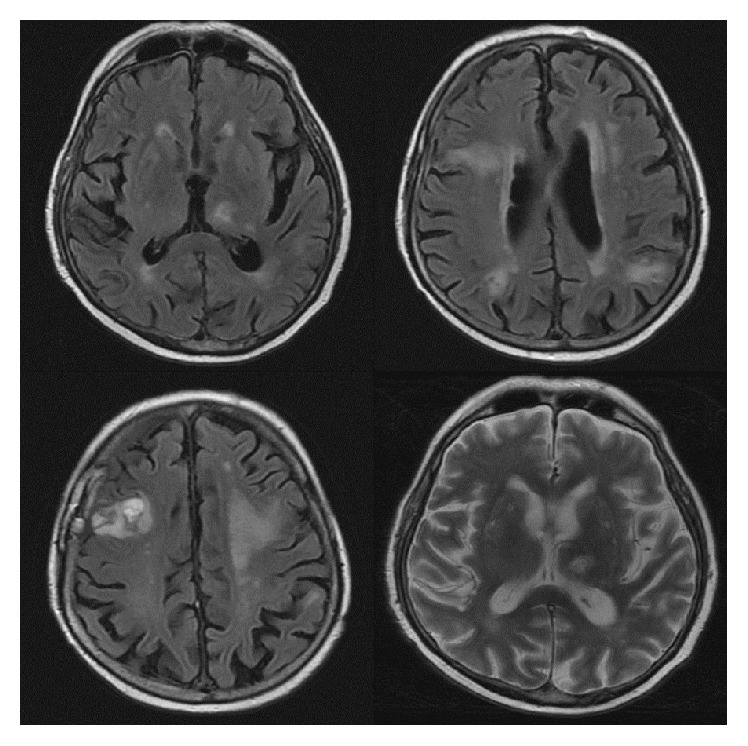
MRI after completion of rituximab and whole brain radiation (day 97). Rituximab started on day 63; cranial XRT started on day 70. Abnormal parenchymal enhancement in both cerebral hemispheres that have decreased in size. Increased hemorrhage in bilateral frontal and parietal lobe lesions. In the right frontal lobe there is a 15 mm rim-enhancing fluid collection. The edema surrounding most of the lesions has decreased in size. The left cerebellar lesion has decreased from 5 mm to 2 mm.

**Figure 6:**
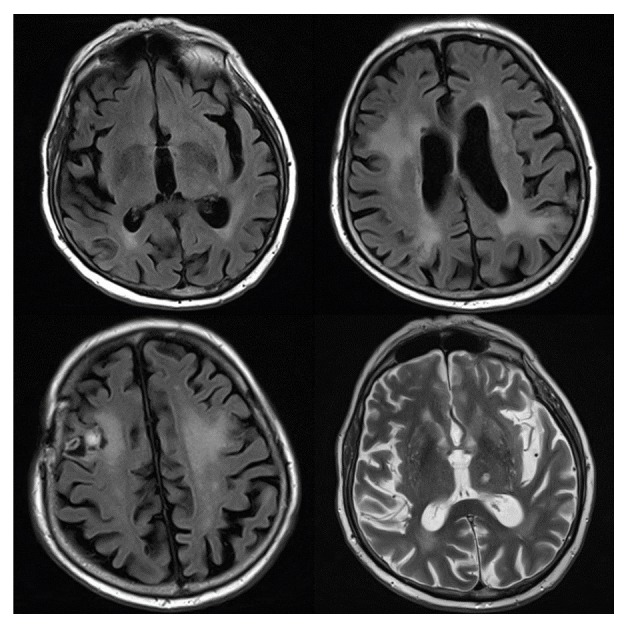
MRI at six-month posttherapy follow-up (day 270). Many of the areas previously demonstrating diffusion hyperintensity have improved and/or resolved. Several areas of diffusion hyperintensity remain. FLAIR hyperintensities are grossly similar except for some improvement in the left thalamic region and some worsening in the right parietal region.

Based on literature review, the decision was made to manage the patient with reduction in immunosuppression, rituximab therapy, and whole brain radiation treatments/cranial radiotherapy (XRT). Mycophenolate mofetil was discontinued; tacrolimus was dosed twice a day following daily levels; prednisone dose was variable. Patient received four cycles at one week intervals of rituximab, starting on day 63 from initial seizure, and five days per week for three weeks of cranial XRT for a total of 30 Gy, starting on day 70 from initial seizure. Following treatment, he had demonstrated marked improvement in his neurologic function; he was following simple commands, able to stand with assistance, and had improvement of hemiparesis with some remnant unilateral weakness. His MRI showed remarkable response with regression of most of the central nervous system lesions (Figure 5). Ultimately, he was discharged to inpatient rehabilitation facility. At one-month follow-up, patient continued to demonstrate neurologic improvements. His MRI six-month posttreatment (Figure 6) showed no increase in lesion size and moderate improvement with resolution of several areas.

## 4. Discussion

PTLD remains a common, morbid complication of solid organ transplantation, yet there is not a standardized therapy or treatment strategy. As PTLD became a more recognized diagnosis, the initial recommendations for therapy were focused on reduction in immunosuppression (RI) and restoration of patients' immune system [8]. In 2006, discussion moved towards treating PTLD that had failed RI with rituximab, an anti-DC-20 monoclonal antibody, or CHOP (cyclophosphamide, doxorubicin, vincristine, and prednisone) chemotherapy. Both treatments were effective, while rituximab was well tolerated and chemotherapy had significant toxicities [9]. Further studies suggested that early treatment with rituximab was associated with improved prognosis [10].

Complicating the treatment modalities of PTLD with CNS involvement is the presence of the blood-brain barrier. Rituximab had shown its effectiveness in systemic PTLD, but the large molecule does not easily pass the blood-brain barrier [4, 7]. In 2012, Yaginuma reports successful treatment of CNS PTLD with whole brain radiation and discontinuation of immunosuppression therapy [7]. Another case report published in the* Journal of Neuro-Oncology* in 2011 [3] demonstrates effective treatment of monomorphic, multifocal, CD20-positive, primary B-cell CNS PTLD with high-dose intravenous rituximab. At one year, MRI showed complete disease resolution, further suggesting a role for early rituximab treatment in CNS PTLD [4]. Given the rarity of primary CNS PTLD, a review of the literature shows a few scattered case reports and series [3, 4, 6, 7, 11, 12] until 2013, when Evens et al. [5] published a multicenter, international analysis of 84 cases of primary CNS PTLD. Treatment options included individual or combinations of rituximab, methotrexate, cytarabine, and whole brain radiation/cranial radiotherapy (XRT). There was no consensus on the optimal treatment, though rituximab and/or cytarabine trended towards improved progression free survival. The most significant finding was a lack of responses to first-line therapy which was a poor prognosticator [5]. In 2014, Tse et al. report complete resolution of CNS PTLD following treatment with rituximab and whole brain radiation [13]. Current accepted treatment modalities include reduction in immunosuppression, chemotherapy, rituximab, whole brain radiation, and surgery.

Prognosis of PTLD with central nervous system involvement is guarded at best. Furthermore, CNS involvement presents with nonspecific symptoms, such as headache, nausea, vomiting, and drowsiness, delaying the diagnosis. As the lesions expand, seizures and neuropsychiatric and focal symptoms are more common and lead to brain imaging [2]. Imaging often demonstrates multifocal, ring-enhancing lesions with a differential diagnosis for infection versus malignancy. Diagnosis is generally based on brain biopsies, typically demonstrating B-cell, EBV positive monomorphic B-cell lymphoma, EBV-associated lymphoma. As the brain lesions tend towards cerebral hemisphere involvement, they are often not conducive to easy biopsy as in the case of our patient. When the lesions are confined to the central nervous system, there is a subsequent delay in proceeding with a brain biopsy, thus delaying diagnosis and therapeutic intervention.

Our patient had a unique presentation of PTLD, with not only rare isolated CNS involvement and beyond median SOT to diagnosis, but also CNS polymorphic variant. Our patient received four cycles of rituximab in conjunction with three weeks of whole brain radiation with marked improvement in both his neuroimaging and his neurologic function. There is no standardization for treatment of primary central nervous system PTLD, but there are clear roles for rituximab and whole brain radiation in addition to reduction in immunosuppression. Furthermore, this case presentation demonstrates a role for continued surveillance and suspicion for a delayed and unusual presentation of PTLD in solid organ transplant patients. Our patient showed neurologically clinical improvement after treatment with rituximab and whole brain radiation; it is our hope that he continues to improve and that this can be used as a model for treatment of other patients with CNS PTLD.

## Figures and Tables

**Figure 1:**
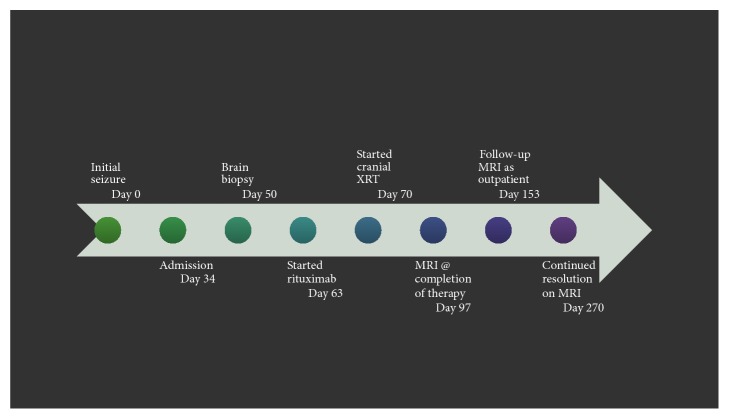
Timeline of events.

**Table 1:** Lab values/infectious work-up.

Serum	
Toxoplasma IgG	14.0 IU/mL
Toxoplasma IgM	Not detected

CSF	

Color	Clear
Opening pressure	18 cmH_2_O
Cell count	100
Lymphocytes	92%
Monocytes	8%
Glucose	85 mg/dL (40–70)
Protein	93 mg/dL (15–45)
West Nile PCR	Negative
VDRL	Nonreactive
VZV IgM	0
Cryptococcus Ag	Negative
CMV PCR	Not detected
EBV PCR	700 u/mL
HSV 1, 2 DNA	Not detected
HSV 1, 2 PCR	Not detected
HHV6 PCR	Not detected
JC virus PCR	Not detected
WBC	9.4 k/cumm
Hemoglobin	11.7 GM/dL
Hematocrit	37.30%
Platelets	251 k/cumm
Sodium	132 mmol/L
Potassium	4.1 mmol/L
Chloride	95 mmol/L
Carbon dioxide	20 mmol/L
BUN	19 mg/dL
Creatinine	1.86 mg/dL
Glucose	209 mg/dL
Calcium	9.6 mg/dL
PT	11.1 sec
INR	1.02
aPTT	29.9 sec
T Bili	0.5 mg/dL
AST	41 units/L
ALT	21 units/L

## Abbreviations

PTLD: Posttransplantation lymphoproliferative disorder
EBV: Epstein-Barr Virus
SOT: Solid organ transplant
RI: Reduction in immunosuppression
MRI: Magnetic resonance imaging
CNS: Central nervous system
PCNS: Primary central nervous system
PET: Positron emission tomography
CT: Computed topography
CHOP: Cyclophosphamide, doxorubicin hydrochloride, vincristine (oncovin), and prednisone chemotherapy
XRT: Radiotherapy.
